# *Metschnikowia pulcherrima* Selected Strain for Ethanol Reduction in Wine: Influence of Cell Immobilization and Aeration Condition

**DOI:** 10.3390/foods8090378

**Published:** 2019-09-01

**Authors:** Laura Canonico, Francesca Comitini, Maurizio Ciani

**Affiliations:** Dipartimento di Scienze della Vita e dell’Ambiente, Università Politecnica delle Marche, Via Brecce Bianche, 60131 Ancona, Italy

**Keywords:** wine, ethanol reduction, *Metschnikowia pulcherrima*, aerated fermentation, immobilized cells

## Abstract

One of the most important problems in the winemaking field is the increase of ethanol content in wine. Wines with high ethanol level negatively affect wine flavor and human health. In this study, we evaluated the use of a selected strain of *Metschnikowia pulcherrima* in immobilized form and under different aeration conditions, to reduce the ethanol content evaluating the volatile profile of the resulting wines. In a preliminary screening the best conditions regarding free/immobilized cells, static/aerated fermentation and inoculation level were identified. Bench-Top fermentation trials with different aeration conditions showed that the use of *M. pulcherrima* selected strain with aeration flow of 20 mL/L/min during the first 72 h of fermentation, led an ethanol reduction of 1.38% (*v/v*) in comparison with *Saccharomyces cerevisiae* control strain. The analytical profile of the resulting wines did not show any negative feature. Indeed, the concentration of ethyl acetate, that above its sensory threshold impacts negatively the wine sensory profile, was found at an acceptable level. On the other hand, an increase in the concentration of significant fruity and flower compounds was found.

## 1. Introduction

Today there is an increasing interest toward the reduction of ethanol content in wines. Indeed, the impact of climate change upon the global production of grapes and the current demand of well-structured and high phenolic content wines determine a generalized increase of ethanol concentration of wines [1,2,3]. Among the various methodologies proposed for the reduction of alcohol content in wine, the microbiological approach seems quite promising to avoid negative variations to the final wine composition [4]. In this regard, *Saccharomyces cerevisiae*, the main yeast species responsible for alcoholic fermentation during winemaking, is not the best yeast species for reducing alcohol levels in wine [5]. Indeed, in general, *S. cerevisiae* commercial starter strains showed similar high ethanol yields, so the researchers focused their attention on the development of new *S. cerevisiae* strains with modified fermentative pathway to obtain wines with reduced alcohol content [6,7,8,9]. On the other hand, non-*Saccharomyces* yeast species have shown great potential to produce less ethanol content in wine [10,11,12]. Indeed, non-*Saccharomyces* yeast can divert carbon away from ethanol production affecting ethanol yield, fermentation efficiency, biomass production and final by-products. In addition, respiro-fermentative regulatory mechanisms exhibited by some non-*Saccharomyces* yeasts (Crabtree negative) are different from that exhibited by *S. cerevisiae* (Crabtree positive). This metabolic behaviour can be exploited to reduce ethanol production through partial and controlled aeration of the grape juice [2,11,13,14]. A previous work [12] showed that the use of immobilized selected strains of *Starmerella bombicola*, *Metschnikowia pulcherrima, Hanseniaspora osmophila* and *Hanseniaspora uvarum* in sequential fermentation with *S. cerevisiae*, could be a suitable strategy to reduce the ethanol content in wine. Sequential fermentation allows to exploit the metabolism of non-*Saccharomyces* yeast while the immobilization procedure allows for high density of cells. In particular, immobilized cells of *S. bombicola* and *M. pulcherrima* under anaerobic conditions led an ethanol reduction of 1.6% and 1.4% respectively, exhibiting an increase of some key aroma compounds. Immobilized cells allow a large amount of cells in confined condition determining high reaction rate, specific physiological conditions and possible reuse. Recently, the use of non-*Saccharomyces* belonging to *M. pulcherrima*, *Torulaspora delbrueckii* and *Zygosaccharomyces bailii* species was investigated, at different aeration conditions to obtain wines with reduced ethanol content but with valuable volatile profile [15].

In the present work we evaluated the use of *M. pulcherrima* in sequential fermentations (*M. pulcherrima/S. cerevisiae*) at different aeration conditions to reduce the alcohol content and maintaining, at the same time, a good aromatic profile of wine. After a preliminary screening to optimize the modalities of inoculum and the effect of aeration, bench-top fermentation trials were carried out. The ethanol reduction and analytical composition of the final wines were evaluated.

## 2. Materials and Methods

### 2.1. Yeast Strains

*M. pulcherrima* DiSVA 269 used in this study was obtained from the Yeast Collection of Department of Life and Environmental Sciences (DiSVA) of Polytechnic University of Marche (Italy) and it was previously selected and evaluated in sequential fermentation trials in immobilized form and free cells under different aeration conditions [12,15]. This yeast was used in sequential fermentation trials with *S. cerevisiae* commercial strain Lalvin EC1118 (Lallemand Inc., Toulouse, France). *S. cerevisiae* strain was also used in pure culture as control. These strains were maintained on Yeast extract–Peptone–Dextrose (YPD) agar medium (Oxoid, Basingstoke, UK) at 25 °C for 48–72 h, and then stored at 4 °C.

### 2.2. Biomass Production and Immobilization Procedures

The biomass for free and immobilized trials was obtained using Modified YPD medium (0.5% yeast extract, 0.1% peptone, 2% dextrose, all *w/v*). *M. pulcherrima* cells were incubated at 25 °C for 72 h in a rotary shaker (150 rpm). The biomass for cell immobilization (5% wet *w/v*) was mixed with 2.5% Na-alginate (Carlo Erba, Milan, Italy) following the procedures described in Canonico et al. [12].

### 2.3. Screening Optimization Cell Concentration on Synthetic Grape Juice (SGJ)

A screening to optimize the immobilized cell concentration of *M. pulcherrima* was carried out in SGJ prepared following the procedures of Ciani and Ferraro [16] and Canonico et al. [12]. Briefly, SGJ was prepared using three different solutions: solution A (sugars); solution B, (tartaric acid, malic acid and citric acid); solution C (vitamins and survival factors). The three solutions were sterilized separately and then combined aseptically. The fermentation trials were set up in 100 mL flask that contained 70 mL SGJ under static and a rotary shaker (150 rpm) at 22 °C in triplicate. The inoculum for the immobilized cells of *M. pulcherrima* was 10%, 5%, 1% of beads (wet *w/v*) which corresponds to an inoculum of 1 × 10^8^ cells/mL, 5 × 10^7^ cells/mL and 1 × 10^7^ cells/mL respectively. The same inoculum was carried out using free cells of *M. pulcherrima*. After 72 h of fermentation, free *S. cerevisiae* cells (1 × 10^6^ cell/mL) were inoculated into the partially fermented grape juice without the removal of free or immobilized cells. At the end of fermentation, fermented products were subjected to chemical and microbiological analysis. The cell concentration trial that exhibited the best ethanol reduction was chosen to carry out fermentation in Natural Grape Juice (NGJ).

### 2.4. Fermentation Trials on Natural Grape Juice (NGJ)

For the fermentation trials in NGJ it was used sterile-filtered (membrane Ø = 0.45 µm) Verdicchio grape juice, an autochthonous white grape variety of Marche region (central Italy). Grape juice was obtained during 2015 vintage and maintained frozen until the use. The main characteristics of the grape juice were: pH 3.39; total acidity 4.42 g/L; total SO_2_, 34 mg/L; malic acid, 2.7 g/L; initial sugar content, 204 g/L; yeast assimilable nitrogen, 90 mg N/L.

Immobilized *M. pulcherrima* cells were evaluated in sequential fermentation trials on Verdicchio grape juice (initial inoculation level 5 × 10^7^ cell/mL selected by the previous screening). After 72 h of fermentation, free *S. cerevisiae* cells (1 × 10^6^ cell/mL) were inoculated into the partially fermented grape juice without removal of free or immobilized cells of *M. pulcherrima*.

The fermentations were carried out in 2 L bench-top bioreactor (Biostat^®^ B; B. *Braun* Biotech Int., Goettingen, Germany) with 1.5 L of Verdicchio grape juice in gentle agitation (60 rpm/min) at 22 °C with the following aeration conditions during the initial 72 h: (i) no aeration; (ii) aeration flow of 1 mL/L/min; (iii) 20 mL/L/min. The inoculation level was 5 × 10^7^ cell/mL of immobilized cells (corresponding to 5% wet *w/v* of beads). Control trials were carried out using free *S. cerevisiae* (10^6^ cell/mL) under no aeration condition. The fermentation kinetics was determined by the evolution of sugar consumption using a specific enzymatic kit (Megazyme International Wicklow Ireland).

The cell release was analyzed by colony forming unit (CFU) counts on Lysine Agar (Oxoid, Hampshire, UK), a selective medium that does not support the growth of *S. cerevisiae* [17]. The fermentations were carried out in triplicate.

### 2.5. Analytical Procedures

The Official European Union Methods [18] were used to determine the ethanol content and volatile acidity g acetic acid/L). Acetaldehyde, ethyl acetate, n-propanol, isobutanol, amyl and isoamyl alcohols were quantified by direct injection into a gas-liquid chromatography system (GC-2014; Shimadzu, Kjoto, Japan). Each sample was prepared and analysed following the procedures of Canonico et al. [19]. Briefly, the main volatile compounds were determined by Solid-phase microextraction (HS-SPME) method. Five mL of each sample was placed in vial containing 1 g NaCl closed with a septum-type cap. HS-SPME was carried out under magnetic stirring for 10 min at 25 °C. After this period, an amount of 3-octanol as the internal standard (1.6 mg/L) was added and the solution was heated to 40 °C and extracted with a fiber Divinylbenzene/Carboxen/Polydimethylsiloxane (DVB/CAR/PDMS) fibre (Sigma-Aldrich, St. Louis, Missouri, USA ) for 30 min by insertion into the vial headspace. The compounds were desorbed by inserting the fibre into a Shimadzu gas chromatograph GC injector for 5 min. A glass capillary column was used: 0.25 μm Supelcowax 10 (length, 60 m; internal diameter, 0.32 mm). The fibre was inserted in split–splitless mode. The compounds were identified and quantified by comparisons with external calibration curves for each compound. The glucose and fructose (K-FRUGL), glycerol (K-GCROL), and succinic acid (K-SUCC) concentrations were determined using specific enzyme kits (Megazyme International, Wicklow Ireland).

### 2.6. Statistical Analysis

Analysis of variance (ANOVA) was applied to the experimental data for the analytical characters of wine. The means were analyzed using the STATISTICA 7 software (Stat Soft, Inc, Tulsa, OK, USA). The significant differences were determined using Duncan tests, and the data were considered significant if the associated *p*-values were <0.05.

## 3. Results

### 3.1. Screening to Assess Aeration, Different Modalities of Immobilization and Inoculation Levels of M. pulcherrima Sequential Fermentation in Synthetic Grape Juice (SGJ)

The preliminary screening was carried out under static and agitation conditions (150 rpm) in SGJ at 22 °C ± 1 °C and inoculated with *M. pulcherrima* (free and immobilized cells), at concentration 10^8^ cell/mL (10% wet *w/v* of beads), 5 × 10^7^ cell/mL (5% wet *w/v* of beads) and 10^7^ cell/mL (1% wet *w/v* of beads). After 72 h of fermentation, *S. cerevisiae* EC 1118 was inoculated (10^6^ cells/mL). The results of the main oenological parameter are shown in Table 1.

*M. pulcherrima/S. cerevisiae* sequential fermentation reduced the ethanol content in almost all conditions tested. *M. pulcherrima* in immobilized form improved the ethanol reduction in comparison with free cells in both static and agitated fermentation conditions. Agitation condition determined a further reduction in ethanol content. A significant ethanol reduction was obtained with 10% and 5% of *M. pulcherrima* immobilized cells both in static and agitation condition while 1 × 10^7^ (1% immobilized cells) did not show significant reduction in comparison with free cells and *S. cerevisiae* control strain. Regarding the volatile acidity, only *M. pulcherrima* immobilized cells (10%) in agitation condition exhibited a significant increase in comparison with the other fermentation trials. Considering the results obtained in this preliminary screening, *M. pulcherrima* at inoculum level 5% corresponding to 5 × 10^7^ cell/mL (the lowest active concentration) was selected for bench-top fermentation trials in NGJ using the following different fermentation conditions: semianaerobic condition (gently agitation 60 rpm), aeration flow of 1 mL/L/min and 20 mL/L/min.

### 3.2. Bench-Top Fermentation Trials

#### 3.2.1. Sugar Consumption in Natural Grape Juice (NGJ) and the Main Analytical Characters

**Figure 1:**
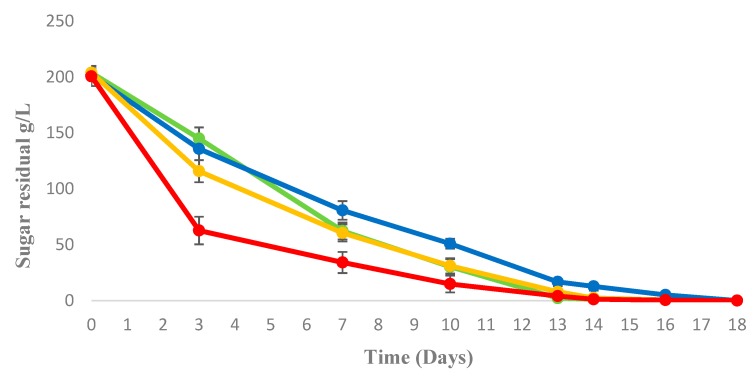
Sugar consumption in sequential fermentation trials of *M. pulcherrima/S. cerevisiae:* semianaerobic (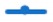); aeration 1 mL/L (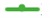); aeration 20 mL/L (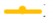); and *S. cerevisiae* pure culture in semianaerobic (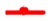).

Figure 1 shows the sugar consumption of trials in different fermentation conditions.

At 72 h of fermentation, before the *S. cerevisiae* inoculum, *M. pulcherrima* with an aeration of 20 mL/L/min, exhibited a higher sugar consumption in comparison with the other sequential fermentation trials. On the other hand, at this time *S. cerevisiae* pure culture exhibited the faster sugar consumption in comparison with all the other trials. During the whole fermentation process, the sequential fermentation trials with 20 mL/L/min and 1 mL/L/min exhibited an intermediate consumption between *S. cerevisiae* pure culture and *M. pulcherrima/S. cerevisiae* in semianaerobic sequential fermentation that was the slowest fermentation. However, all sequential fermentations completely consumed the initial sugar content in grape juice and *M. pulcherrima* cell release from beads was lower than 10^3^ cell/mL in all trials.

The main analytical characters of *M. pulcherrima* sequential fermentations under different aeration conditions at 72 h (before inoculum of *S. cerevisiae*) and at the end of fermentation are reported in Table 2.

Results of volatile acidity and glycerol content at 72 h did not show significant differences unlike ethanol content that showed significant differences among the trials. *S. cerevisiae* pure cultures displayed the highest production while the ethanol formation by *M. pulcherrima* was related to aeration indicating that the oxygen positively affects the fermentation rate of *M. pulcherrima*.

*M. pulcherrima* sequential fermentation with aeration flow of 1 mL/L/min and 20 mL/L/min increased significantly the glycerol content in the final wine in comparison with semianaerobic fermentation trials (*M. pulcherrima/S. cerevisiae* and *S. cerevisiae* pure culture). Similarly, to 72 h of fermentation, acetic acid content of final wines showed a comparable value among the trials.

The results of the final ethanol content highlighted a relevant differentiation among the fermentation trials. In the condition tested in semianaerobic condition *M. pulcherrima/S. cerevisiae*, there was an ethanol reduction of 0.51% (*v/v*) in comparison with *S. cerevisiae* control trial. This value was comparable with the ethanol reduction obtained with *M. pulcherrima* sequential fermentation with an aeration flow of 1mL/L/min. A significant increase of ethanol reduction of ca. 1.30% (*v/v*) was obtained with *M. pulcherrima* sequential fermentation with an aeration flow 20 mL/L/min. The data of ethanol yield reflected followed this trend.

#### 3.2.2. The Main By-Products and Volatile Compounds of Sequential Fermentation with *M. pulcherrima* in Different Aeration Conditions in NGJ

To evaluate the influence of different aeration conditions on the aromatic profile of wines, the main volatile compounds produced in NGJ were assayed and the results were reported in Table 3.

A significant increase in ethyl acetate content in fermentation carried out with *M. pulcherrima* with aeration flow of 20 mL/L/min was found. However, differently from the results shown in similar conditions (sequential fermentation and aeration flow during the first 72 h) using free cells [15], the increase of ethyl acetate with immobilized cells was restrained and under the negative sensory threshold for this compound (175 mg/L) [20]. Moreover, this condition (immobilized cells and aeration flow of 20 mL/L/min) determined a significant increase of desirable volatile compounds such as ethyl butyrate and isoamyl acetate. In semianerobic condition, *M. pulcherrima* sequential fermentation exhibited a significantly higher amount of phenyl ethyl acetate in comparison with the other trials. Regarding the main higher alcohols *M. pulcherrima* sequential fermentation with aeration flow 1 mL/L/min exhibited a significant increase in amylic and isoamylic alchols, while with aeration flow 20 mL/L/min increased significantly the production of β-phenyl ethanol and isobutanol respectively. A significant increase of acetaldehyde content showed sequential fermentations under semianaerobic condition and 20 mL/L/min of air flow. However, acetaldehyde at these concentrations did not negatively influence the aromatic profile of the final wines. Regarding the carboxylic acids, a significant increase was found only for the diethyl succinate content in the trial with 20 mL/L/min of air flow, while no significant differences were exhibited in butyric acid content.

## 4. Discussion

One of the main issues related to winemaking is the progressive increase of the ethanol content in wine. The microbiological approaches seem an attractive strategy to reduce alcohol production [21]. Some of these strategies are focused on *S. cerevisiae* yeast genetically modified [6,7,8,22], evolution-based strategies, together with breeding strategies [9,23] while others focused the attention on the use of non-*Saccharomyces* wine yeast [13,21,24,25]. Here we evaluated the effect of different aeration condition and cell immobilization on *M. pulcherrima* sequential fermentation with *S. cerevisiae* on ethanol reduction and volatile profile of wines. In a previous study, the use of different non-*Saccharomyces* yeasts in immobilized form in anaerobic condition was investigated [12]. In particular, in that work the same strain of *M. pulcherrima* used in the present study, led an ethanol reduction of 1.4% (*v/v*) using 10% (*w/w*) of beads corresponding to an inoculation level of 10^8^ cells /mL. In another recent work, using *M. pulcherrima* sequential fermentation (free cell inoculation) under aeration condition, a reduction of 1.6% (*v/v*) of ethanol content was obtained, however, this produced an excessive amount of ethyl acetate [15]. The addition of oxygen during the early stage of fermentation stimulates wine fermentation, favoring fermentation activity by promoting sterol and unsaturated fatty acids (UFAs) biosynthesis [26,27]. Results of this work confirm the helpful action of the oxygen on yeast metabolism improving the fermentation activity of *M. pulcherrima* immobilized cells highlighted by an increased sugar utilization kinetics.

In this work, the results of the ethanol content highlighted that the combined use of immobilized cells at reduced inoculation level (5% (wet *w/v*) of beads) and aeration flow 20 mL/L/min produced a relevant ethanol reduction (1.38% (*v/v)*) without the formation of ethyl acetate at an unacceptable level as free cells [15]. On the other hand, the amount of ethyl acetate formed is still relevant and could be controlled, optimizing the air flow during the first stage of fermentation.

Ethyl acetate is one of the main esters produced during wine fermentation, and while low concentrations impart a fruity aroma (below 80 mg/L), at concentrations above 160–170 mg/L give undesirable ‘nail polish remover’ and ‘solvent’ sensory descriptors [20]. On the other hand, some relevant aroma compounds such as ethyl butyrate and isoamyl acetate increased. This aspect is related to the oxygen supplementation as reported by Valero et al. [28] and Shekhawat et al. [29] that showed an increase in the concentration of esters and higher alcohols. Relative to the use of oxygen during wine fermentation, there are different results regarding its impact on different aromatic compounds. These controversial results could be due to the use of different strains, grape must and fermentation conditions [8,28,29,30].

Another important aspect is the reduction of the inoculum of immobilized cells. Indeed, in comparison to the previous work [12], it was halving the inoculum (5% (wet *w/w*) instead of 10% (wet *w/w*) of beads) that considerably reduced the use costs of this application, while maintaining a relevant reduction of ethanol content.

Regarding the acetic acid content, non-*Saccharomyces* yeasts generally produce an increase of acetic acid. This compound responsible for sour and bitter taste which is at higher levels of 0.9 g/L is considered detrimental for wine quality [11,14,20,29]. Rocker et al. [14] evaluated different non-*Saccharomyces* strains under anaerobic conditions and found the resulting wines with ‘vinegar’ sensory descriptor. In this study, *M. pulcherrima* tested in different fermentation conditions exhibited an acetic acid content comparable to the one exhibited by *S. cerevisiae*.

In summary, our results confirm the ability of non-*Saccharomyces* yeasts to produce wines with reduced ethanol content but it is necessary to set up the modalities of their use in function of the physiological and fermentation characteristics of the specie/strain. Here, we obtained a relevant reduction of ethanol and a valuable volatile composition of wines modulating oxygen supplied during the early stages of fermentation using the immobilized cells.

## Figures and Tables

**Table 1:** Main fermentation parameters of preliminary screening of *M. pulcherrima* in sequential fermentation trials with *S. cerevisiae* EC1118 inoculated after 72 h of fermentation in SGJ.

Fermentation Trials. (Agitation Condition)	Sugar Consumed (g/L)	Ethanol (%*v/v*)	Volatile Acidity (g/L)	Fermentation Trials (Static condition)	Sugar Consumed (g/L)	Ethanol (%*v/v*)	Volatile Acidity (g/L)
*S. cerevisiae*	219.8 ± 0.1 ^a^	12.49 ± 0.19 ^a^	0.45 ± 0.03 ^bc^	*S. cerevisiae*	219.9 ± 1.6 ^a^	12.63 ± 0.12 ^ab^	0.42 ± 0.01 ^ab^
*M. pulcherrima* free cells (10%)	219.9 ± 0 ^a^	12.15 ± 0.21 ^ab^	0.40 ± 0.08 ^bc^	*M. pulcherrima* free cells (10%)	219.9 ± 2 ^a^	12.42 ± 0.19 ^ab^	0.27 ± 0.01 ^b^
*M. pulcherrima* free cells (5%)	219.9 ± 0 ^a^	12.08 ± 0.08 ^ab^	0.45 ± 0.06 ^bc^	*M. pulcherrima* free cells (5%)	219.9 ± 1.5 ^a^	12.57 ± 0.14 ^ab^	0.44 ± 0.05 ^ab^
*M. pulcherrima* free cells (1%)	219.9 ± 0 ^a^	12.31 ± 0.36 ^ab^	0.50 ± 0.00 ^b^	*M. pulcherrima* free cells (1%)	219.7 ± 0.3 ^a^	12.72 ± 0.02 ^a^	0.39 ± 0.05 ^ab^
*M. pulcherrima* immobilized cells (10%)	219.9 ± 0 ^a^	11.87 ± 0.11 ^b^	0.60 ± 0.00 ^a^	*M. pulcherrima* immobilized cells (10%)	219.9 ± 1.3 ^a^	11.92 ± 0.02 ^c^	0.43 ± 0.07 ^ab^
*M. pulcherrima* immobilized cells (5%)	219.9 ± 0 ^a^	11.95 ± 0.04 ^b^	0.36 ± 0.00 ^c^	*M. pulcherrima* immobilized cells (5%)	219.9 ± 0.6 ^a^	12.38 ± 0.05 ^b^	0.39 ± 0.05 ^ab^
*M. pulcherrima* immobilized cells (1%)	219.8 ± 0.1 ^a^	12.07 ± 0.03 ^ab^	0.45 ± 0.00 ^ab^	*M. pulcherrima* immobilized cells (1%)	219.8 ± 0.5 ^a^	12.33 ± 0.04 ^ab^	0.50 ± 0.03 ^a^

The initial sugar concentration was 220 g/L. Data are means ± standard deviations from three independent experiments. Data with different superscript letters (^a,b,c^) within each column are different homogeneous groups according to Duncan tests (0.05%).

**Table 2:** Main analytical characters of sequential fermentation with *M. pulcherrima* in different fermentation conditions semianaerobic, 1 mL/L/min and 20 mL/L/min at 72 h and at the end of fermentation.

72 h of Fermentation	Glycerol (g/L)	Acetic Acid (g/L)	Ethanol (% *v/v*)	Ethanol Yield (g/g)	End of Fermentation	Glycerol (g/L)	Acetic Acid (g/L)	Ethanol (%*v/v*)	Ethanol Yield (g/g)
*M. pulcherrima * 1 mL/L/min	0.41±0.01 ^b^	0.35 ± 0.00 ^a^	4.24 ± 0.15 ^c^	33.4 ± 0.13 ^b^	*M. pulcherrima * 1 mL/L/min	7.04 ± 0.14 ^a^	0.33 ± 0.03 ^b^	11.28 ± 0.14 ^b^	43.8 ± 0.16 ^c^
*M. pulcherrima * 20 mL/L/min	0.41 ± 0.01 ^b^	0.34 ± 0.00 ^a^	6.20 ± 0.12 ^b^	37.7 ± 0.18 ^a^	*M. pulcherrima * 20 mL/L/min	7.12 ± 0.03 ^a^	0.36 ± 0.02 ^b^	10.62 ± 0.12 ^c^	41.2 ± 0.14 ^d^
*M. pulcherrima* semianaerobic	0.46 ± 0.04 ^b^	0.30 ± 0.00 ^b^	1.97 ± 0.18 ^d^	30.4 ± 0.14 ^c^	*M. pulcherrima* semianaerobic	5.43 ± 0.09 ^b^	0.36 ± 0.02 ^b^	11.49 ± 0.08 ^b^	44.5 ± 0.04 ^b^
*S. cerevisiae* semianaerobic	0.47 ± 0.01 ^a^	0.33 ± 0.00 ^a^	8.65 ± 0.06 ^a^	38.6 ± 0.13 ^a^	*S. cerevisiae* semianaerobic	5.15 ± 0.06 ^b^	0.38 ± 0.02 ^b^	12.00 ± 0.01 ^a^	46.5 ± 0.01 ^a^

Data are means ± standard deviations from three independent experiments. Data with different superscript letters (^a,b,c^) within each column are different homogeneous groups according to Duncan tests (0.05%).

**Table 3:** Main volatile compounds of sequential fermentation with *M. pulcherrima* in different aeration conditions (mg/L).

Fermentation Trials	*M. pulcherrima * 1 mL/L/min	*M. pulcherrima * 20 mL/L/min	*M. pulcherrima* Semianaerobic	*S. cerevisiae* Semianaerobic
**Esters**				
Ethyl butyrate	0.12 ± 0.06 ^c^	0.96 ± 0.004 ^a^	0.575 ± 0.03 ^b^	0.14 ± 0.01 ^c^
Isoamyl acetate	0.25 ± 0.07 ^b^	1.67 ± 0.01 ^a^	0.240 ± 1.14 ^b^	0.136 ± 0.04 ^c^
Ethyl hexanoate	0.05 ± 0.02 ^b^	0.15 ± 0.09 ^a^	0.079 ± 0.05 ^b^	0.062 ± 0.02 ^b^
Phenyl ethyl acetate	0.021 ± 0.005 ^a^	0.01 ± 0.0006 ^a^	0.031 ± 0.020 ^a^	0.02 ± 0.002 ^a^
Ethyl acetate	46.3 ± 3.6 ^b^	169 ± 5.4 ^a^	37 ± 5.9 ^c^	23.00 ± 2.07 ^d^
**Alcohols**				
β-Phenyl ethanol	18.52 ± 0.10 ^c^	30.42 ± 0.44 ^a^	26.20 ± 0.07 ^b^	9.655 ± 0.19 ^d^
n-Propanol	18.9 ± 0.4 ^b^	21.54 ± 1.74 ^a^	20.04 ± 1.89 ^a^	18.3 ± 2.0 ^b^
Isobutanol	58.9 ± 4.0 ^b^	120.50 ± 4.21 ^a^	42.13 ± 2.00 ^c^	14.9 ± 3.7 ^d^
Amylic alcohol	34 ± 5.64 ^a^	27.02 ± 1.17 ^b^	18.13 ± 3.45 ^c^	20.1 ± 0.4 ^c^
Isoamylic alcohol	241.6 ± 21.2 ^a^	181.81 ± 6.45 ^b^	164.3 ± 16.84 ^c^	159.61 ± 4.21 ^c^
**Carboxylic Acids**				
Butyric acid	0.024 ± 0.003 ^a^	0.083 ± 0.06 ^a^	0.038 ± 0.006 ^a^	0.022 ± 0.002 ^a^
Diethyl succinate	0.002 ± 0.002 ^b^	0.024 ± 0.02 ^a^	0.008 ± 0.0 30 ^ab^	0.005 ± 0.000 ^b^
**Monoterpens**				
Linalool	0.011 ± 0.003 ^a^	0.004 ± 0.01 ^b^	0.004 ± 0.002 ^b^	0.000 ± 0.001 ^c^
Geraniol	0.009 ± 0.003 ^b^	0.122 ± 0.003 ^ab^	0.006 ± 0.08 ^b^	0.232 ± 0.003 ^a^
**Carbonyl Compounds**				
Acetaldehyde	14.91 ± 2.57 ^b^	50.52 ± 4.37 ^a^	66.9 ± 18.7 ^a^	22.50 ± 4.74 ^b^

Data are means ± standard deviations from three independent experiments. Data with different superscript letters (^a,b,c^) within each row, are different homogeneous groups according to Duncan tests (0.05%).
